# Profiling of transcriptional and epigenetic changes during directed endothelial differentiation of human embryonic stem cells identifies FOXA2 as a marker of early mesoderm commitment

**DOI:** 10.1186/scrt192

**Published:** 2013-04-24

**Authors:** Lynsey Howard, Ruth M Mackenzie, Nikolay A Pchelintsev, Tony McBryan, John D McClure, Martin W McBride, Nicole M Kane, Peter D Adams, Graeme Milligan, Andrew H Baker

**Affiliations:** 1 British Heart Foundation Glasgow Cardiovascular Research Centre, Institute of Cardiovascular and Medical Sciences, 126 University Place, Glasgow, UK G12 8TA; 2 Institute of Cancer Sciences, Garscube Estate, Beatson Institute for Cancer Research, College of Medical, Veterinary and Life Sciences, University of Glasgow, Glasgow, UK, G61 1BD; 3 Institute of Neuroscience and Psychology, 58 Hillhead Street, College of Medical, Veterinary and Life Sciences, University of Glasgow, Glasgow, UK G12 8QB

**Keywords:** Gene expression, Epigenetics, Endothelial differentiation, Embryonic stem cells, Transcription factors

## Abstract

**Introduction:**

Differentiation of vascular endothelial cells (ECs) in clinically relevant numbers for injection into ischaemic areas could offer therapeutic potential in the treatment of cardiovascular conditions, including myocardial infarction, peripheral vascular disease and stroke. While we and others have demonstrated successful generation of functional endothelial-like cells from human embryonic stem cells (hESCs), little is understood regarding the complex transcriptional and epigenetic changes that occur during differentiation, in particular during early commitment to a mesodermal lineage.

**Methods:**

We performed the first gene expression microarray study of hESCs undergoing directed differentiation to ECs using a monolayer-based, feeder-free and serum-free protocol. Microarray results were confirmed by quantitative RT-PCR and immunocytochemistry, and chromatin immunoprecipitation (ChIP)-PCR analysis was utilised to determine the bivalent status of differentially expressed genes.

**Results:**

We identified 22 transcription factors specific to early mesoderm commitment. Among these factors, FOXA2 was observed to be the most significantly differentially expressed at the hESC–EC day 2 timepoint. ChIP-PCR analysis revealed that the *FOXA2* transcription start site is bivalently marked with histone modifications for both gene activation (H3K4me3) and repression (H3K27me3) in hESCs, suggesting the transcription factor may be a key regulator of hESC differentiation.

**Conclusion:**

This enhanced knowledge of the lineage commitment process will help improve the design of directed differentiation protocols, increasing the yield of endothelial-like cells for regenerative medicine therapies in cardiovascular disease.

## Introduction

The directed differentiation of human embryonic stem cells (hESCs) towards endothelial cell (EC) lineages offers therapeutic potential in the treatment of myocardial infarction, peripheral vascular disease and stroke [1]. While successful derivation of endothelial-like cells from hESCs has been demonstrated [2-5], relatively little is understood regarding early commitment to mesoderm and subsequent specification [6].

To commit to a specified lineage, pluripotent cells must undergo radical transcriptional change [2], partly regulated by miRNAs [2,3]. Recent studies suggest epigenetic influences also play a significant role in the determination of cell fate [7]. Indeed, the poised transcriptional state classically associated with pluripotent hESCs is maintained via effects at the chromatin level with gene expression determined by post-translational modification of histones [8]. While some histone marks, such as trimethylation of lysine 4 of histone H3 (H3K4me3), are associated with gene activation, others, such as trimethylation of lysine 27 of histone H3 (H3K27me3), are associated with repression. However, around 3,000 developmental regulatory genes in embryonic stem cells are labelled with both H3K4me3 and H3K27me3 [7], allowing the genes to be rapidly activated upon differentiation or to remain silenced during commitment to lineages not requiring their expression. Unsurprisingly, these bivalently marked genes are proposed to be master regulators of the differentiation process, although their role in endothelial differentiation has not been extensively investigated to date.

The study described herein was designed to profile transcriptional and epigenetic changes during early hESC commitment to a mesodermal and endothelial-like fate with a view to improving understanding of this process and to optimise the generation of ECs for regenerative medicine purposes.

## Methods

### Cell culture

hESC lines SA461 (Cellartis, Dundee, UK), H1 and H9 (WiCell Research Institute, Madison, WI, USA) and RC10 (Roslin Cells Ltd, Edinburgh, UK) were cultured in a monolayer-based, serum-free and feeder-free system. Pluripotency was maintained and endothelial differentiation induced as previously described [2]. Primary human saphenous vein endothelial cells (HSVECs) were isolated on the day of surgery by standard collagenase digestion based on a modified version of the protocol described by Jaffe and colleagues [9]. HSVECs were then cultured as previously described [2]. All participants gave written informed consent. The study was approved by the West of Scotland Ethics Committee (06/S0703/110) and complies with the principles of the Declaration of Helsinki.

### Microarray analysis

**Figure 1:**
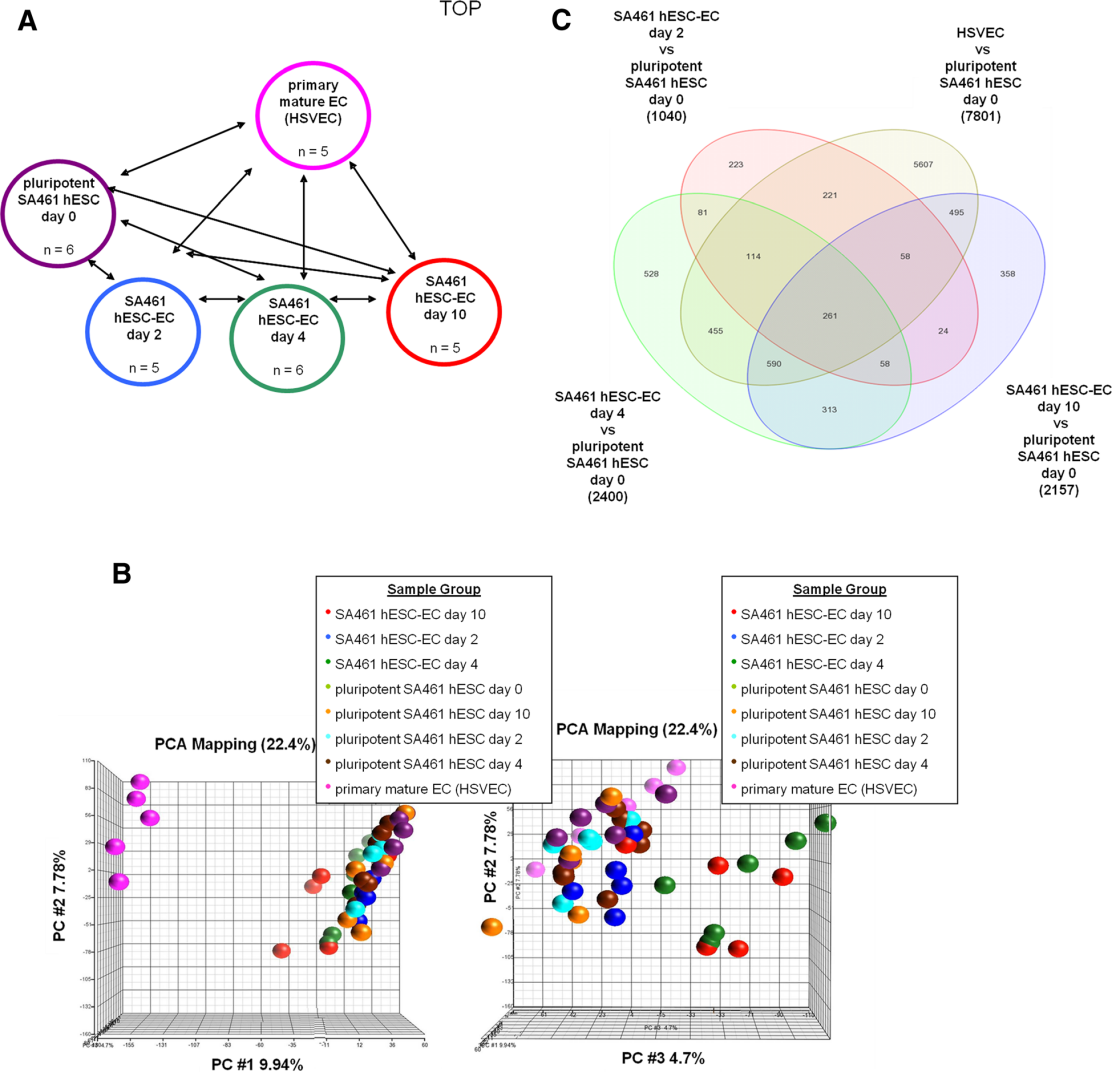
**Illumina ® (Saffron Walden, UK) whole genome expression microarray. (A)** Study design with different timepoints under directed human embryonic stem cell (hESC)–endothelial cell (EC) differentiation. Arrows represent pairwise comparisons performed. **(B)** Principle component analysis (PCA) showing separation of primary mature human saphenous vein endothelial cells (HSVECs) and hESC-derived cells. **(C)** Summary of differentially expressed probe sets. Numbers in brackets represent total number of differentially expressed probe sets for the associated pairwise comparison.

SA461 hESCs subjected to directed endothelial differentiation were harvested at days 0, 2, 4 and 10, and RNA was isolated using the miRNAeasy Mini Kit (Qiagen, Crawley, UK). HSVEC RNA was harvested to act as a positive control. Complementary RNA was prepared for hybridisation with Illumina® HT-12 v3 Expression BeadChip microarrays (Illumina®, Saffron Walden, UK) and the gene expression assessed. Microarray data were deposited in the ArrayExpress public repository [ArrayExpress: E-MTAB-1510]. Figure 1A outlines the experimental design.

Data were quantile normalised and background subtracted in Genome Studio (Illumina®) and were then exported to Partek® Genomics Suite™ (Partek® Inc., St. Louis, MO, USA) and visualised using principle component analysis [10]. An error-weighted analysis of variance with a false discovery rate multiple testing adjustment threshold of 0.05 was used to identify differentially expressed probe sets [11], which were then uploaded to Ingenuity Pathway Analysis software (2009; Ingenuity® Systems, Redwood City, CA, USA) and analysed to identify dynamic gene expression.

### *In silico* prediction of bivalency

H3K27me3 and H3K4me3 data from the H9 chromatin immunoprecipitation (ChIP)-sequencing dataset of Ku and colleagues [12] were mined and integrated with microarray data to give an *in silico* prediction of the bivalent status of genes.

SICER software (http://home.gwu.edu/~wpeng/Software.htm) was used to determine enriched domains for each histone modification using a stringent E value of 0.1, a window size of 200 and gap sizes of 200, 400, 600, 800 and 1,000 base pairs [13]. For each gene within the Ensembl gene set (NCBI36.1), the transcription start site (TSS) was determined and an interval spanning ±2 kb around the TSS was defined.

For a specific gap size, if the TSS contained enriched domains for both H3K27me3 and H3K4me3 marks, then this gene was classified as bivalent. The bivalency score was defined as the count of gap sizes where a gene was considered bivalent. A score of 0 would thus indicate that the gene was never classified as bivalent, while a score of 5 indicated that the gene was found to be bivalent regardless of the gap size chosen.

### TaqMan® quantitative RT-PCR

First-strand cDNA was synthesised from 1 μg DNase-treated total RNA using TaqMan Reverse Transcription Reagents (Applied Biosystems, Life Technologies Ltd., Paisley, UK). TaqMan® Gene Expression Assays for *CXXC1* (Hs00969406_g1), *EHMT2* (Hs00938384_g1), *FOXA2* (Hs00232764_m1), *L3MBTL2* (Hs01002038_g1), *MLL3* (Hs01005539_m1), *RBM14* (Hs01056358_m1), *TAF6L* (Hs01008038_m1), *TFAP4* (Hs01558245_m1), *TSC22D3* (Hs00608272_m1), *UBTF* (Hs00610733_g1), *USF2* (Hs01100995_g1) and *ZNF35* (Hs01071488_m1) were used with TaqMan® Endogenous Controls, *18S* (Hs99999901_s1) or *UBC* (Hs00824723_m1) (Applied Biosystems, Life Technologies). Relative quantitation of gene expression was calculated using the comparative (ΔΔCt) method [14].

### Immunocytofluorescence

Immunocytofluorescence experiments were carried out as previously described [2]. Primary antibodies utilised were mouse anti-OCT4 primary antibody (SC5279, 1:200; Santa Cruz Biotechnology Inc., Dallas, TX, USA) and goat anti-FOXA2 primary antibody (AF2400, 1:50; R&D Systems Europe Ltd., Abingdon, UK). Secondary antibodies were Alexafluor-488 donkey anti-goat (A11055; Invitrogen, Life Technologies Ltd., Paisley, UK) and Alexafluor-555 goat anti-mouse (A21424; Invitrogen). ProLong Gold with 4′,6-diamidino-2-phenylindole (Invitrogen) was used for nuclear counterstaining.

### Chromatin immunoprecipitation and PCR identification

ChIP assays were performed based on a modified version of the method of Rai and colleagues [15]. Chromatin was prepared from pluripotent H9s and SA461s, and H3K4me3 and H3K27me3 were immunoprecipitated using Dynabeads® M-280 sheep anti-rabbit IgG (Invitrogen) and H3K4me3 (AB8580; Abcam, Cambridge, UK) and H3K27me3 (C36B11; Cell Signaling Technology, Beverly, MA, USA) specific antibodies. Immunoprecipitations with total H3 (AB1791; Abcam) and control IgG (M7023; Sigma-Aldrich Company Ltd., Dorset, UK) were included as positive and negative controls, respectively.

Using the University of California Santa Cruz Genome Browser, primer pairs were designed to span the *FOXA2* TSS (Additional files 1, 2 and 3) and DyNAmo™ SYBR® Green quantitative PCR (Thermo Fisher Scientific UK Ltd., Loughborough, UK) was performed on immunoprecipitation eluates, in addition to 2% chromatin input not subjected to immunoprecipitation. Quantitative PCR data were normalised to IgG negative control and displayed as fold enrichment.

### Statistical analyses

Values are presented as mean ± standard error of the mean. Data from multiple groups were analysed using repeated-measures analysis of variance. Significant differences were determined by Tukey *post-hoc* testing and *P* <0.05 (two-tailed) was considered significant.

## Results

### Gene expression analysis of endothelial differentiation

Principle component analysis of global transcription data, designed to detect early transcriptional changes during directed differentiation (Figure 1A), revealed sufficient separation of cell groups. As expected, HSVECs were clearly distinct from all hESC-derived cells, and hESC–EC on day 4 and day 10 were divergent compared with day 0 and day 2 timepoints (Figure 1B).

A large number of significantly differentially expressed probe sets was observed at each of the three differentiation timepoints, as compared with day 0 (Figure 1C). Comparison of samples from day 10 hESC–EC with HSVEC samples revealed differential expression of 6,133 different probe-sets, defining markedly different cells (Figure 1C).

Further investigation of the hESC–EC day 2 timepoint was carried out and 223 significantly differentially expressed, unique probe sets were identified (Figure 1C). We reasoned that corresponding genes might be involved in early differentiation. The Ingenuity Pathway Analysis software revealed that these probe sets correspond to 178 genes (Additional file 4), a relatively high proportion (>12%) of which encode for transcription factors (Table 1).

**Table 1:** Differentially expressed human embryonic stem cell–endothelial cell day 2 transcription factor-encoding genes

**Gene symbol**	**Entrez gene name**	**Fold change**	**FDR**	**Bivalency score**
ANKRA2	Ankyrin repeat, family A (RFXANK-like), 2	−1.64	0.01	0
ASCL2	Achaete-scute complex homolog 2 (Drosophila)	1.24	0.05	5
CXXC1	CXXC finger protein 1	1.82	0.02	0
DNMT3L	DNA (cytosine-5)-methyltransferase 3-like	1.29	0.01	0
EHMT2	Euchromatic histone-lysine *N*-methyltransferase 2	1.46	0.02	0
FOXA2	Forkhead box A2	1.51	<0.005	5
GABPB1	GA binding protein transcription factor, beta subunit 1	−1.47	0.02	0
HDAC5	Histone deacetylase 5	1.21	0.03	0
L3MBTL2	l(3)mbt-like 2 (Drosophila)	1.37	0.03	0
MLL3	Myeloid/lymphoid or mixed-lineage leukemia 3	1.28	0.01	0
MNT	MAX binding protein	1.50	0.02	0
RAX2 (human)	Retina and anterior neural fold homeobox 2	1.57	0.04	0
RBM14	RNA binding motif protein 14	1.72	0.01	0
RFX1 (includes EG:100038773)	Regulatory factor X, 1 (influences HLA class II expression)	1.47	0.02	0
SNAPC4	Small nuclear RNA activating complex, polypeptide 4, 190 kDa	1.80	0.04	0
SRA1	Steroid receptor RNA activator 1	1.34	0.01	0
TAF6L	TAF6-like RNA polymerase II, p300/CBP-associated factor-associated factor, 65 kDa	1.75	0.01	0
TFAP4	Transcription factor AP-4 (activating enhancer binding protein 4)	1.62	0.01	0
TSC22D3	TSC22 domain family, member 3	−1.73	<0.005	5
UBTF	Upstream binding transcription factor, RNA polymerase I	1.60	0.02	0
USF2	Upstream transcription factor 2, c-fos interacting	1.73	0.04	0
ZNF35	Zinc finger protein 35	1.33	0.02	0

Fold change and false discovery rate (FDR) for the 22 differentially expressed genes are shown. *In silico* prediction of bivalency involved assigning bivalency scores from 0 to 5: 0, not bivalently marked; 5, genes are robustly bivalent with enriched domains of H3K4me3 and H3K27me3 within 2 kb of their transcription start site.

**Figure 2:**
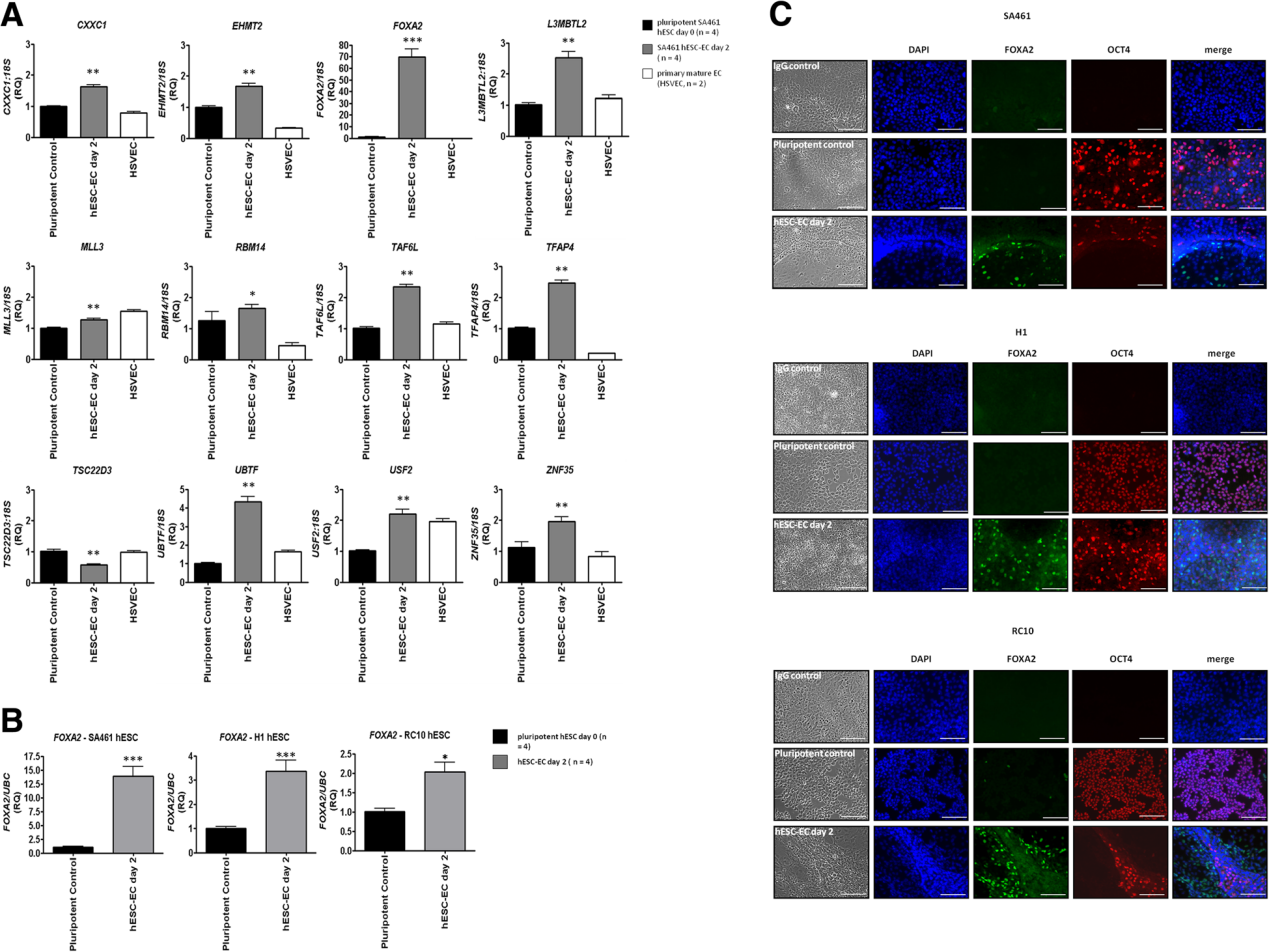
**Validation of human embryonic stem cell–endothelial cell day 2 transcription factor differential expression. (A)** Differential mRNA expression of 12 of the 22 transcription factors was confirmed by TaqMan® quantitative RT-PCR. **(B)** Differential expression of *FOXA2* mRNA was confirmed in H1s and RC10s by TaqMan® quantitative RT-PCR. *P <0.05, **P <0.01 and ***P <0.001 versus pluripotent control. RQ, relative quantitation. **(C)** Immunocytofluorescent staining showing upregulation of FOXA2 protein expression at human embryonic stem cell–endothelial cell (hESC–SC) day 2. DAPI, 4′,6-diamidino-2-phenylindole; HSVEC, primary human saphenous vein endothelial cell. Scale bars = 20 μm.

To validate this differential expression of transcription factors, quantitative RT-PCR was performed. Of the 22 genes listed, 12 were confirmed as being differentially expressed – including *FOXA2*, which demonstrated the most significant and rapid upregulation of all genes examined (Figure 2A). Upregulation of *FOXA2* at hESC–EC day 2 was also confirmed in additional H1 and RC10 hESC lines (Figure 2B). Again, upregulation was transient as analysis of day 4, day 7 and day 10 differentiations failed to detect expression of *FOXA2* mRNA (data not shown).

To confirm day 2 differential expression of FOXA2 at the protein level, SA461s, H1s and RC10s were stained using anti-FOXA2 antibodies. Expression of the pluripotency marker OCT4 was observed in day 0 pluripotent cells but diminished by hESC–EC day 2 (Figure 2C). FOXA2, although present in a subset of pluripotent cells, was abundantly expressed at day 2. Interestingly, FOXA2 did not co-localise with OCT4 (Figure 2C).

### Epigenetic analysis of *FOXA2*

On aligning *in silico* data with differentially expressed genes identified via microarray analysis, a large proportion (2,684/3,883) achieved a bivalency score of 5 and was therefore predicted to be bivalently marked.

**Figure 3:**
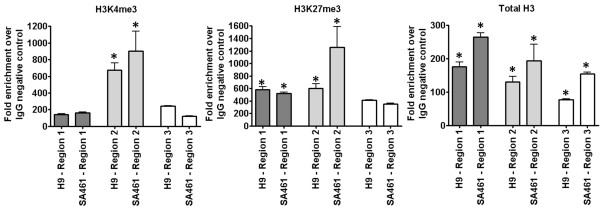
**In vitro validation of predicted *FOXA2 *bivalency.** Immunoprecipitation of H3K4me3, H3K27me3 and total H3 from chromatin of pluripotent SA461s and H9s revealed enrichment of the histone modifications at the *FOXA2* transcriptional start site, as identified by quantitative PCR using primers for three regions, covering around 2.5 kb in total. *P <0.05 versus IgG control.

With epigenetics known to influence cell fate, the predicted bivalent status of genes encoding hESC–EC day 2 differentially expressed transcription factors was of particular interest. *FOXA2* was identified as having both H3K4me3 and H3K27me3 modifications at its TSS (Table 1) and ChIP-PCR confirmed bivalency of the *FOXA2* TSS in pluripotent H9s and SA461s (Figure 3).

## Discussion

To the best of our knowledge, this study describes the first global gene expression profiling of hESCs at intermediate timepoints during their directed differentiation to an endothelial-like lineage using a monolayer-based, feeder-free and serum-free protocol. The study is also the first to identify transcription factors potentially specific to early mesoderm commitment, including FOXA2 which we show to be bivalently marked at the TSS. In addition, we report that cells at day 10 of the differentiation process remain genetically distinct from mature endothelial cells, supporting our previous findings that this timepoint yields progenitor-type cells, capable of further differentiation *in vivo*[2].

Rapid and transient *FOXA2* mRNA upregulation was identified and confirmed at the protein level, suggesting the hepatocyte nuclear factor may represent a marker of early mesoderm lineage commitment. A member of the forkhead class of DNA binding proteins, FOXA2 has been shown to bind and open compacted chromatin at histones H3 and H4 [16] and is one of the first transcription factors to bind target genes on differentiation [17]. Traditionally endoderm associated, FOXA2 has not, as far as we are aware, previously been linked to hESC–EC differentiation. However, with cells used for microarray analyses likely to represent a somewhat heterogeneous population containing precursors for both endoderm and mesoderm germ layers [18,19], further investigation utilising purified cell populations will be required to confirm the importance of FOXA2 in early EC commitment.

Epigenetic control of transcription is now known to play a major role in cell fate determination and, as stated, we have identified the *FOXA2* TSS as being bivalently marked in pluripotent hESCs, displaying histone modifications associated with both gene activation and repression. Conceivably, FOXA2 may therefore be a marker of early mesoderm lineage commitment and a potential regulator of hESC-EC commitment. Such enhanced knowledge of the complex commitment process is likely to prove important for optimising the design and development of directed endothelial differentiation protocols.

## Conclusion

This study is the first to generate global gene expression profiles for hESCs undergoing directed differentiation to ECs using a monolayer-based, serum-free and feeder-free system, and is the first to identify transcription factors, including FOXA2, likely to be specific for early mesoderm lineage commitment.

## Abbreviations

ChIP: Chromatin immunoprecipitation; EC: Endothelial cell; H3K27me3: Histone H3 trimethylated on lysine 27; H3K4me3: Histone H3 trimethylated on lysine 4; hESC: Human embryonic stem cell; HSVEC: Primary human saphenous vein endothelial cell; miRNA: microRNA; PCR: Polymerase chain reaction; RT: Reverse transcription; TSS: Transcription start site.

## Competing interests

The authors declare that they have no competing interests.

## Authors’ contributions

LH, RMM and NAP performed experimental studies, and analysed and interpreted data. JDM and MWM contributed to the design of the microarray study and performed analysis of microarray data. TM generated bivalency predictions. AHB, GM, PDA and NMK conceived of the study and participated in its design and coordination. The manuscript was drafted by RMM with critical input from all authors. All authors read and approved the final manuscript.

## Supplementary Material

Additional file 1 **A figure showing the University of California Santa Cruz Genome Browser visualisation of H3K4me3 and H3K27me3 ChIP sequencing performed on pluripotent H9 hESCs.** Genome browser output from *FOXA2* genomic location showing presence of H3K4me3 and H3K27me3 at the transcriptional start site. Output is based on the pluripotent H9 hESC ChIP sequencing data of Ku and colleagues [12]. Click here for file

Additional file 2 **A figure showing the location of primer pairs designed for *FOXA2 *ChIP-PCR.** University of California Santa Cruz Genome Browser output showing the genomic location of the optimised primer pairs designed for the *FOXA2* transcription start site. Click here for file

Additional file 3 **A table presenting the sequences of forward and reverse primers used for *FOXA2 *** **ChIP-PCR.** Click here for file

Additional file 4 **A table presenting the differentially expressed genes identified on Ingenuity Pathway Analysis software of Partek® Genomics Suite™ hESC–EC day 2 microarray data.** Within the hESC–EC day 2 specific dataset, Ingenuity Pathway Analysis software identified 178 genes as being differentially expressed (*P* ≤0.05). Click here for file
