# Supplementary figures and images for: Profiling of transcriptional and epigenetic changes during directed endothelial differentiation of human embryonic stem cells identifies FOXA2 as a marker of early mesoderm commitment
Source: Stem Cell Res Ther. 2013 Apr 24;4(2):36. doi: 10.1186/scrt192 (PMC3706826; doi:10.1186/scrt192)

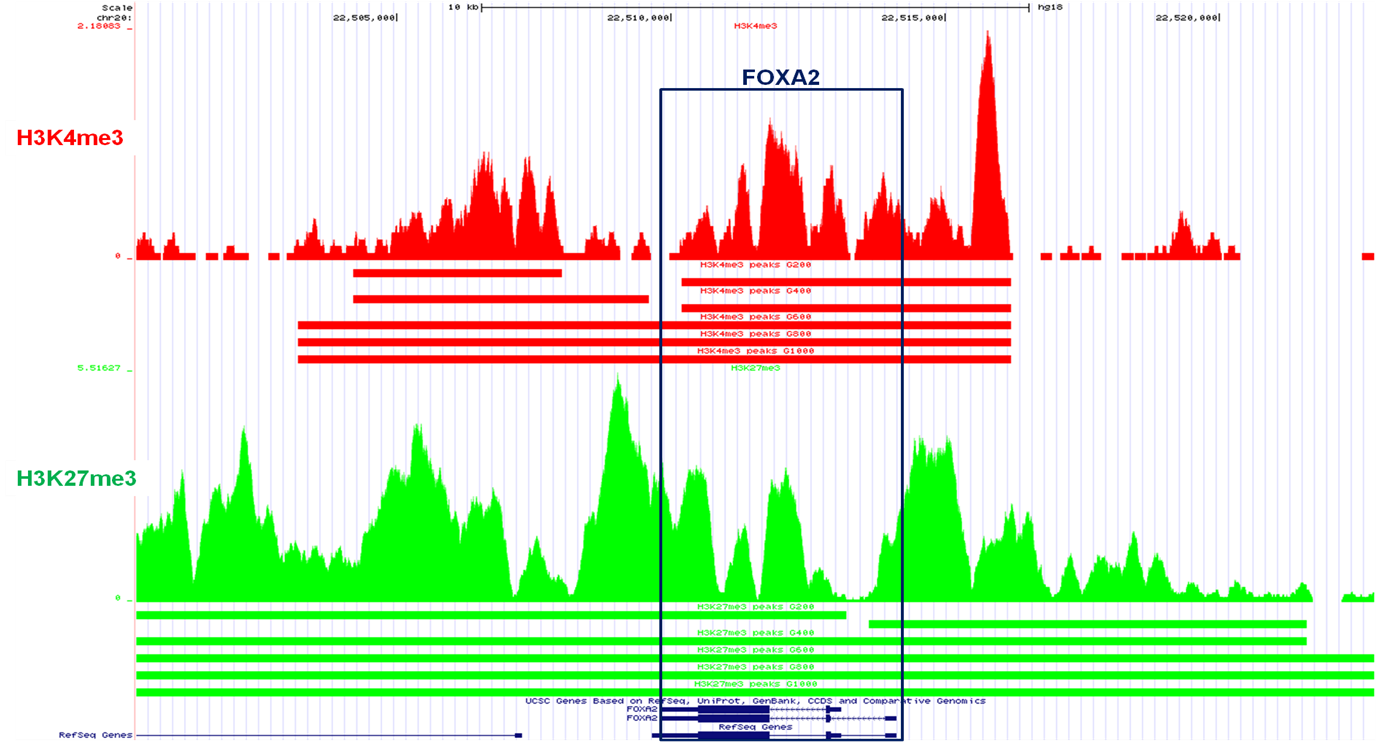

Supplement: Additional file 1 — A figure showing the University of California Santa Cruz Genome Browser visualisation of H3K4me3 and H3K27me3 ChIP sequencing performed on pluripotent H9 hESCs. Genome browser output from FOXA2 genomic location showing presence of H3K4me3 and H3K27me3 at the transcriptional start site. Output is based on the pluripotent H9 hESC ChIP sequencing data of Ku and colleagues [12]. [file scrt192-S1.tiff]

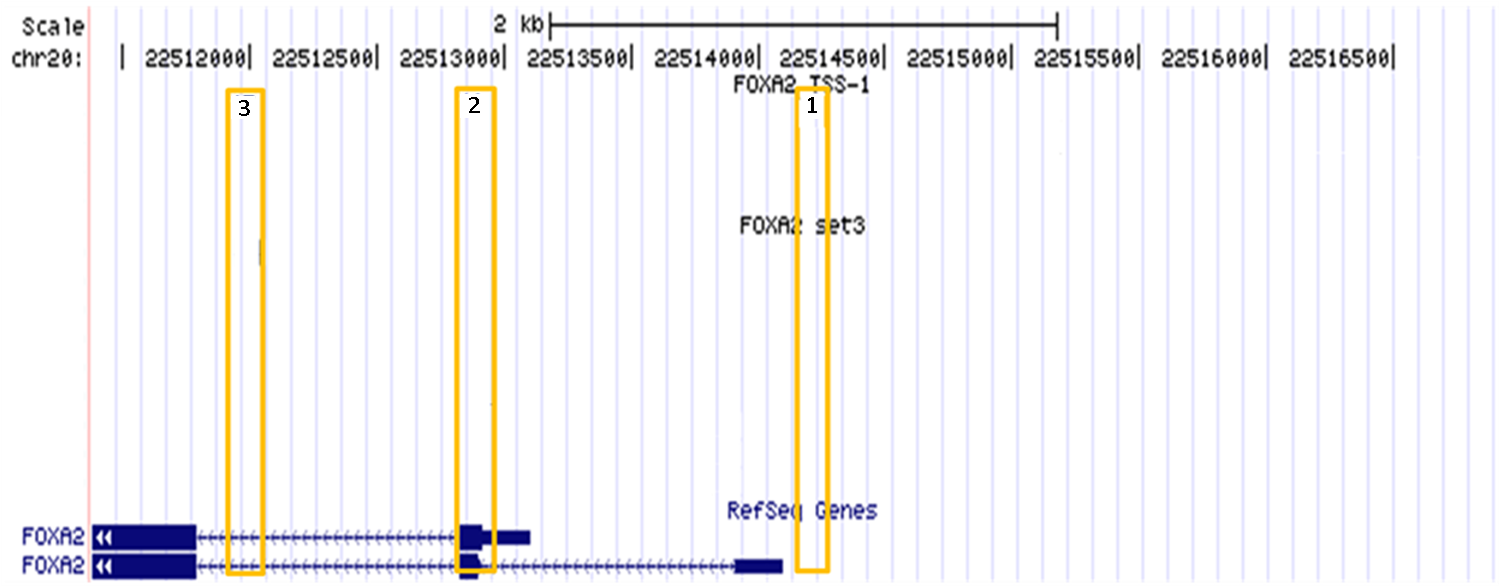

Supplement: Additional file 2 — A figure showing the location of primer pairs designed for FOXA2 ChIP-PCR. University of California Santa Cruz Genome Browser output showing the genomic location of the optimised primer pairs designed for the FOXA2 transcription start site. [file scrt192-S2.tiff]
